# Automated Phenotyping Tool for Identifying Developmental Language Disorder Cases in Health Systems Data (APT-DLD): A New Research Algorithm for Deployment in Large-Scale Electronic Health Record Systems

**DOI:** 10.1044/2020_JSLHR-19-00397

**Published:** 2020-08-11

**Authors:** Courtney E. Walters, Rachana Nitin, Katherine Margulis, Olivia Boorom, Daniel E. Gustavson, Catherine T. Bush, Lea K. Davis, Jennifer E. Below, Nancy J. Cox, Stephen M. Camarata, Reyna L. Gordon

**Affiliations:** a Department of Otolaryngology, Vanderbilt University Medical Center, Nashville, TN; b Neuroscience Program, College of Arts and Science, Vanderbilt University, Nashville, TN; c Vanderbilt Brain Institute, Vanderbilt University, Nashville, TN; d Department of Hearing and Speech Sciences, Vanderbilt University Medical Center, Nashville, TN; e Kennedy Krieger Institute, Baltimore, MD; f Vanderbilt Genetics Institute, Vanderbilt University Medical Center, Nashville, TN; g Department of Medicine, Vanderbilt University Medical Center, Nashville, TN

## Abstract

**Purpose:**

Data mining algorithms using electronic health records (EHRs) are useful in large-scale population-wide studies to classify etiology and comorbidities (Casey et al., 2016). Here, we apply this approach to developmental language disorder (DLD), a prevalent communication disorder whose risk factors and epidemiology remain largely undiscovered.

**Method:**

We first created a reliable system for manually identifying DLD in EHRs based on speech-language pathologist (SLP) diagnostic expertise. We then developed and validated an automated algorithmic procedure, called, Automated Phenotyping Tool for identifying DLD cases in health systems data (APT-DLD), that classifies a DLD status for patients within EHRs on the basis of ICD (International Statistical Classification of Diseases and Related Health Problems) codes. APT-DLD was validated in a discovery sample (*N* = 973) using expert SLP manual phenotype coding as a gold-standard comparison and then applied and further validated in a replication sample of *N* = 13,652 EHRs.

**Results:**

In the discovery sample, the APT-DLD algorithm correctly classified 98% (concordance) of DLD cases in concordance with manually coded records in the training set, indicating that APT-DLD successfully mimics a comprehensive chart review. The output of APT-DLD was also validated in relation to independently conducted SLP clinician coding in a subset of records, with a positive predictive value of 95% of cases correctly classified as DLD. We also applied APT-DLD to the replication sample, where it achieved a positive predictive value of 90% in relation to SLP clinician classification of DLD.

**Conclusions:**

APT-DLD is a reliable, valid, and scalable tool for identifying DLD cohorts in EHRs. This new method has promising public health implications for future large-scale epidemiological investigations of DLD and may inform EHR data mining algorithms for other communication disorders.

**Supplemental Material:**

https://doi.org/10.23641/asha.12753578

A 2016 National Academies report called attention to the urgent need to improve the identification of children with communication impairments and to increase access to treatment (The National Academies of Sciences, Engineering, and Medicine, 2016). In parallel, timely innovations in the use of large-scale health systems data point to an underutilized but potentially data-rich resource for epidemiological approaches that could advance our understanding of speech and language disorders (Raghavan et al., 2018). These recent advances highlight a gap in the use of valuable population data that could be harnessed not only to improve the identification of communication disorders at the population level but also to address the public health consequences of pediatric language-related disorders. More effective use of available public health data linked to biobank repositories may also help identify genetic and other health risk factors linked to language disorders (LDs); the resulting knowledge could pave the way to improved systems of screening, identification, and provision of treatment (Raghavan et al., 2018).

This study is aimed at harnessing information from electronic health record (EHR) databases to reliably identify occurrences of developmental language disorder (DLD) to address the need for better application of health data resources for a prevalent communication disorder. DLD is a common type of communication impairment defined by delay and difficulty in acquiring spoken language, distinct from major developmental disabilities such as autism spectrum disorder (ASD) and intellectual disability that also affect language (American Psychiatric Association, 2013; Bartak et al., 1975; Simms & Jin, 2015). The clinical manifestations of DLD are characterized by disruptions in underlying morphosyntax and/or vocabulary in receptive and/or expressive language domains (Bishop et al., 2016; Lancaster & Camarata, 2019).

Although the etiology of DLD is multifactorial (Bishop et al., 2017; Leonard, 2014a, 2014b), it is defined as atypical language occurring without the precipitation of external trauma, abuse, brain damage, or known neurological pathology (Bishop, 2017; Kamhi, 1998; Stark & Tallal, 1981). Yet, even after ruling out those alternative causes, there are many commonly occurring comorbidities with DLD, including speech disorder, attention-deficit/hyperactivity disorder, dyslexia, as well as delays in learning and scholastic skills (Leonard, 2014b; Pennington & Bishop, 2009; Raghavan et al., 2018). Partly due to the interference of their language difficulties with educational trajectory (Conti-Ramsden et al., 2018; Law et al., 2009), individuals with DLD experience a wide range of long-term negative academic, social, and economic consequences (Eadie et al., 2018; Hubert-Dibon et al., 2016; Law, 2002). Large-scale screening and diagnostic studies indicate that this highly prevalent disorder, which affects an estimated 7% of the population, remains considerably under-identified in school-aged children (Bishop et al., 2017; McLeod & Harrison, 2009; Tomblin et al., 1997).

Recently, there has been debate in the literature regarding the classification of LDs in children (Volkers, 2018a). The *Diagnostic and Statistical Manual of Mental Disorders* (5th ed.; American Psychiatric Association, 2013) uses the term “language disorder,” with “developmental” added herein to distinguish this phenotype from acquired LD, which can occur in childhood secondary to a neurological event such as traumatic brain injury (TBI). DLD is in common use in the literature (see Bishop, 2017). There has also been a common phenotype designated *specific language impairment* (SLI; see Leonard, 2014b, for a review) that has a narrower definition, with a requirement that nonverbal intellectual abilities be within the typical range. However, some scholars use the terms “SLI” and “DLD” interchangeably (e.g., Govindarajan & Paradis, 2019). We decided to use DLD herein because (a) it is a more clinically and educationally applicable classification (Bishop et al., 2017; Raghavan et al., 2018; Volkers, 2018b) and, (b) although both SLI and DLD refer to developmental deficits in the understanding and/or use of language, SLI is a narrower, more restrictive phenotypic definition (normal nonverbal IQ, lack of comorbid developmental or intellectual disorders; Stark & Tallal, 1981; Tomblin et al., 1996), which, many authors have argued, is counterintuitive to the complex etiology accompanying DLDs (Bishop, 2017; Bishop et al., 2017; Conti-Ramsden & Durkin, 2016; Hayiou-Thomas et al., 2014).

The relationship between the language deficits in LDs and general cognitive development is complex and has long been the subject of much inquiry and debate (Camarata & Swisher, 1990). Research suggests that, although cognitive ability is lower in children with DLD than in their typically developed peers (Earle et al., 2017; Plante, 1998), nonverbal intelligence is no longer thought to represent a ceiling for language development (Camarata et al., 2014; Reilly et al., 2014; Rice, 2015). The broader definition of DLD, unlike SLI, focuses on functional language impairment irrespective of deficits in nonverbal IQ, so long as the case does not meet the phenotypic characterization as being globally intellectually disabled (Bouck & Satsangi, 2015; Lancaster & Camarata, 2016; Marrus & Hall, 2017). Much prior research on LDs has focused specifically on the narrower SLI terminology when determining exclusionary criteria; thus, the broader population of children with DLD may not be represented in the aggregate body of research. Finally, SLI is a term not as widely used as DLD in clinical populations (Volkers, 2018b), which could have implications for extracting diagnostic phenotypes from EHRs.

With the recent expansion of the research definition of DLD as described above, identifying DLD cases in existing epidemiological data sets would allow the field to advance our understanding of the underlying biology of DLD in a more clinically relevant representation of its heterogeneity (McGregor et al., 2020; Raghavan et al., 2018). Moreover, such approaches could provide valuable new information about the health, economic, and social effects of DLD (The National Academies of Sciences, Engineering, and Medicine, 2016; Casey et al., 2016) and would complement prior research discovery on the more narrowly, historically defined SLI (see also McGregor et al., 2020, for an in-depth discussion). New knowledge of risk factors from population-level approaches could be rapidly put to use to improve delivery of clinical services, thus addressing the urgent need to close the identification gap and to expand access to speech-language pathology treatment for DLD.

An emerging opportunity for scaling up population-wide research approaches for investigating health conditions and identifying diseases, disorders, medical concerns, treatment, biometrics, and related risks is the utilization of biobanks with EHRs. EHRs are the electronically maintained medical records of patients and include diagnostic codes, billing history, lab results, and other health information. One important component of EHRs is ICD (*International Statistical Classification of Diseases and Related Health Problems*; https://www.cdc.gov/nchs/icd/index.htm) *codes* and medical notes to preserve and track medical history (Wei et al., 2016). In addition, EHRs are sometimes directly linked to biological testing (i.e., lab values, tissue samples, genotypes, or genetic sequencing) of the patient in specialized databases called “biobanks.” Thus, EHRs are potentially a rich source of pertinent phenotypic information and, in some cases, genomic information, which can be used for a wide variety of research purposes (Breitenstein et al., 2018; Pathak et al., 2013; Prieto et al., 2016). Even within de-identified EHRs, automated data mining algorithms can capture patient-specific ICD billing codes, medications, and lab test results to define inclusion and exclusion criteria and identify cases with the phenotype of interest, without disclosing patient ID (Pathak et al., 2013; Pendergrass & Crawford, 2019; Rasmussen-Torvik et al., 2014; Richesson et al., 2016).

This research must proceed with an understanding that DLD is a complex disorder with multiple profiles and possible co-occurring conditions. While recent work has applied automated EHR phenotyping in EHRs to autism and attention-deficit/hyperactivity disorder (Connolly, 2013; Glicksberg et al., 2018; Lingren et al., 2016), communication disorders remain largely underrepresented in this field, and discovery into their genetic basis has lagged behind that of other psychiatric traits. The challenge of accruing large enough sample sizes, or big data, for novel epidemiological discovery (especially into the polygenic architecture of these complex traits; Wray et al., 2008) can be met by using population-scale EHR-linked databases with automated phenotyping algorithms (e.g., Butte & Kohane, 2006; Chen et al., 2018; Esteban et al., 2017; Ludvigsson et al., 2013; Safarova et al., 2016; Smoller, 2018; Zhong et al., 2016). Here, we propose the mobilization of EHRs for automated phenotyping of DLD; this approach has the potential to fill gaps in the literature by identifying associated characteristics, risk factors, comorbidities, underlying etiology, and prognostic indicators related to DLD (Bishop et al., 2016; Dyck et al., 2011; Justice et al., 2019; Lancaster & Camarata, 2019; see the review in Leonard, 2014b; Raghavan et al., 2018).

The goal of this project is thus to develop and evaluate an automated phenotyping algorithm that reliably identifies cohorts of DLD cases from large EHR databases, using a stable and definable DLD phenotype derived from speech-language pathology–informed principles. Moreover, we sought to implement such an algorithm using only the ICD codes of the records and the EHR entry date for LDs, to maximize the portability of the algorithm into other health systems–linked biobank research data that may only contain ICD codes and dates. It is important to note that the development of an EHR DLD phenotype is a process requiring refinement and speech-language pathologist (SLP) expert validation because DLD does not have a unique ICD diagnostic code and is furthermore a phenotype with varied manifestations wherein multiple features of language (vocabulary, grammar, etc.) may be affected. Thus, establishing and validating a robust algorithmic approach to phenotyping DLD in ICD-based EHRs represents significant advancement.

Here, we describe the algorithm we developed and tested, called, Automated Phenotyping Tool for identifying DLD cases in health systems data (APT-DLD), to classify DLD cases within Vanderbilt University Medical Center's (VUMC's) de-identified EHR database. Whereas efficient and holistic diagnosis of DLD by SLPs in the clinical world is, of course, geared toward individual specific therapeutic purposes, the value of APT-DLD is as a research tool that enables the *classification* of DLD cases in EHR databases, allowing the communication disorders, human genetics, and health policy fields to effectively tackle population-level, epidemiological risk factors of DLD (including large-scale genetic inquiry). We hypothesize that this approach could be applied to other large EHR databases with similar ICD codes to identify cases at the population level.

## Method

### Description of the VUMC EHR Database

The broader target population for this technique includes pediatric records from EHR systems with ICD indicators of DLD. In our study, we utilized pediatric data obtained from VUMC's EHR system known as Synthetic Derivative, which currently includes EHRs from over 3.1 million patients (https://sd.app.vumc.org/sddiscover/#) who have received medical attention/treatment at a VUMC-affiliated institution, with each EHR corresponding to a single person's medical records. All Synthetic Derivative records are de-identified according to the Health Insurance Portability and Accountability Act of 1996 regulations to an extent that makes reidentification impossible. Patient information recorded in the EHR database includes a de-identified patient ID; demographic information such as age, sex, and ethnicity; and clinical information including medications, lab values, test results, procedural and surgical codes, and diagnostic codes. ICD 9th edition (ICD-9) and ICD 10th edition (ICD-10) coding systems are used by health care professionals to both classify and code all symptoms, procedures, and diagnoses. Both ICD-9 and updated ICD-10 code sets are present in the Synthetic Derivative database. Each independent visit to the medical center is electronically entered and added to the patient's EHR, thus constituting a longitudinal medical history of that patient. The Synthetic Derivative database is linked to a biobank called “BioVU” (https://victr.vumc.org/what-is-biovu/), where phenotypic information of a subset of patients' EHRs is associated with their biological data. EHRs from the Synthetic Derivative and BioVU databases are primarily used by researchers for cohort identification, phenomic analyses, and genetic analyses (Gill et al., 2019; Karol et al., 2015; Malinowski et al., 2014; Verma et al., 2016; Wei et al., 2014; Wheeler et al., 2017). Ethical use of the data reported in the current article is covered by an approved institutional review board exemption (application no. 150643), and all users working with these data filed data use agreements with the institution that set forth policies and procedures that protect the integrity and confidentiality of the data.

### Study Population and Overview of Approach

Our discovery sample for DLD phenotyping was derived from EHRs found in Synthetic Derivative's biobank-linked patient subset known as BioVU (corresponding to 246,561 EHRs at the time our study sample was extracted). Since future directions for this line of research (outside the scope of this article) include genetic analyses of DLD, we used EHRs from BioVU (from here on referred to as the *discovery database*) for our discovery sample. In addition, the BioVU-linked EHRs tended to have more enriched clinical notes, which were ideal for Step 2 below. A multistep strategy was used to first select the discovery sample from the discovery database and then identify and validate the classification of the DLD cohort, as follows:

1. An (automated) ICD-based broad search was conducted to identify a pediatric discovery sample of records where LD symptoms were present.
2. A manual chart review rubric was developed, refined, and deployed in the discovery sample to distinguish between DLD cases versus other cases with LD symptoms caused by other conditions (i.e., other neurodevelopmental disorders such as ASD or acquired LDs due to stroke or head injury).
3. The APT-DLD algorithm was developed to mimic the classification results of the manual chart review.
4. APT-DLD was refined relative to a gold-standard SLP clinician coder's DLD phenotype coding in the discovery sample and then tested in relation to manual chart review.

**Figure 1.**
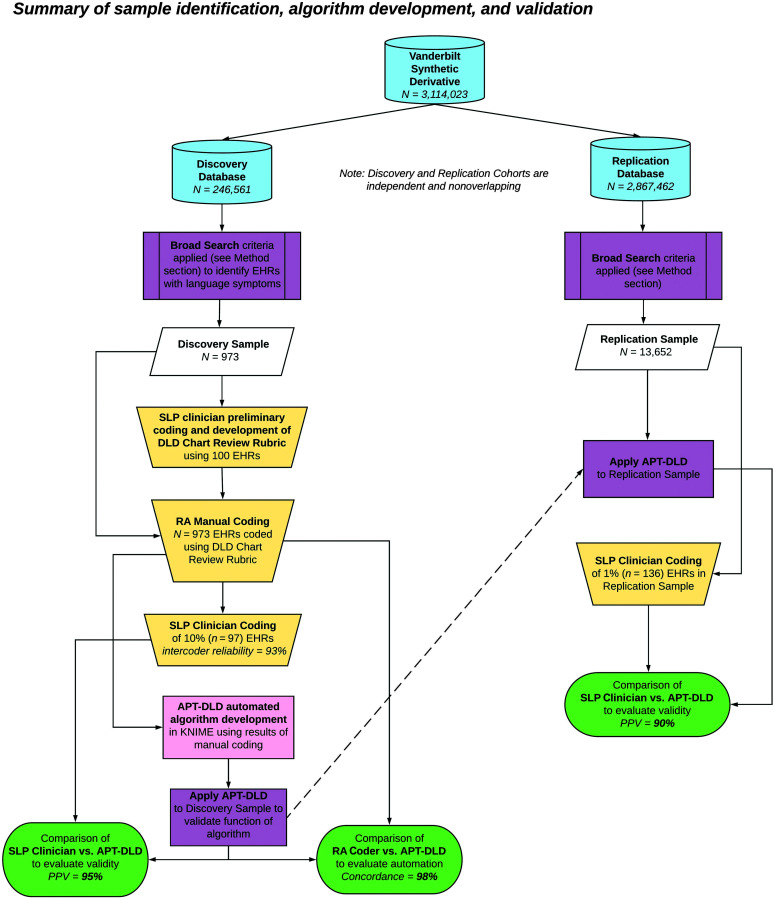
Overview of the process employed for the identification of study samples from extant electronic health record (EHR) research databases, rubric refinement, and development and validation of the Automated Phenotyping Tool for identifying DLD cases in health systems data (APT-DLD) algorithm in the discovery and replication samples. Discovery and replication samples are independent and nonoverlapping. DLD = developmental language disorder; RA = research assistant; SLP = speech-language pathologist.

An overview of the entire process, from identifying samples to manual chart review, coding refinement, and algorithm development, is shown in Figure 1. At the conclusion of algorithm development in the discovery sample, APT-DLD was subsequently deployed and validated in a larger replication sample derived from the replication database, which consisted of EHRs in the Synthetic Derivative database, excluding those records that were already accessed in the discovery database, in order to ensure total independence of the discovery and replication samples.

### Availability of Open-Source Algorithm

**Figure 2.**
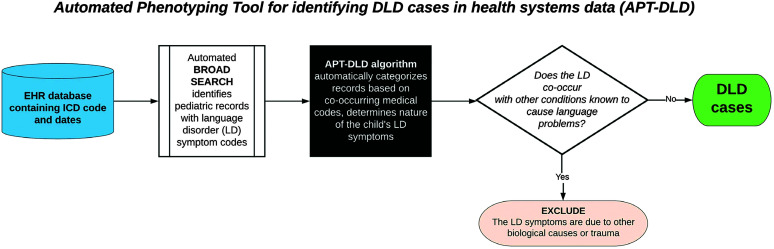
Automated Phenotyping Tool for identifying DLD cases in health systems data (APT-DLD) simple overview. The major components of preparing the data and applying the APT-DLD algorithm are summarized here. Further details on algorithm processes are provided in Figure 4. DLD = developmental language disorder; EHR = electronic health record; ICD = International Statistical Classification of Diseases and Related Health Problems.

Both the broad search and APT-DLD are fully automated, and materials/codes for both components are freely accessible (https://phekb.org/phenotype/developmental-language-disorder), with two implementation options (KNIME and R) on PheKB, a phenotype knowledgebase website that stores electronic algorithms for public use (Kirby et al., 2016). As shown in Figure 2, users will be able to apply both the broad search for the identification of LD cases from an EHR database and APT-DLD to further classify DLD cases as “included” for further study and the remaining LD cases as “excluded.”

### Broad Search (Step 1)

#### Discovery Sample

Utilizing ICD-9 and ICD-10 codes, we applied our initial broad inclusion and exclusion search criteria with an age filter to the discovery database, to identify a pediatric discovery sample of *N* = 973 EHRs that met all the criteria. For the broad search, the inclusion criteria consist of six codes for LD symptoms commonly used clinically by SLPs to diagnose DLD: ICD-9 codes: 315.31 (expressive language disorder), 315.32 (mixed expressive–receptive language disorder), and 315.39 (other developmental speech or language disorders); ICD-10 codes: F80.1 (expressive language disorder), F80.2 (mixed expressive–receptive language disorder), and F80.89 (other developmental disorders of speech and language). The exclusion criteria include codes for hearing loss (ICD-9 code: 389; ICD-10 codes: Group H90 and Group H91), intellectual disabilities (ICD-9 codes: 317, 318, and 319; ICD-10 codes: F70, F71, F72, F73, F78, and F79), and chromosomal abnormalities (ICD-9 code: Group 758; ICD-10 codes: Groups Q90–Q99). Further details regarding inclusion and exclusion criteria are reported in Supplemental Material S1 (Developmental Language Disorder Manual Chart Review Rubric).

The broad search inclusion and exclusion criteria were determined by our research team (including authors K. M., O. B., and S. M. C. who are certified SLPs) to best capture LD symptoms that likely correspond to DLD, because this disorder does not have an exclusive ICD code of its own; rather, these six codes that correspond to various LD symptoms are used to define it clinically. Note that ICD-10 Code F80.9 (developmental disorders of speech and language, unspecified) was not among the broad search inclusion criteria because this code is not often used by SLPs and is usually associated with other neurodevelopmental or physiological disorders (American Speech-Language-Hearing Association, 2019).

Alternate causes of language delay/disorder were ruled out by excluding records that contained codes for hearing loss and intellectual disabilities because these conditions may cause or be associated with LD symptoms but are distinct from DLD (e.g., Bouck & Satsangi, 2015). Chromosomal abnormalities were excluded as well because DLD is a phenotype without an identified genetic etiology (e.g., Down syndrome, Prader–Willi syndrome) even when concomitant LDs are present. It is important to note that ICD codes are not necessarily indicative of a definitive diagnosis (e.g., codes for chromosomal abnormalities might be used to indicate testing for the disorder but not the presence of the disorder) but are still relied upon to indicate medical issues. Keeping this in mind, we understand that our algorithm is conservative in its identification of LDs. Here, *n* = 1,065 records were identified that met the criteria described above; an age filter was then applied to restrict the sample to pediatric patients who had their first LD code occurring before the age of 18 years (individuals with their first LD code occurring after the age of 18 years commonly had language codes due to neurological conditions and/or trauma events and were thus excluded from our sample). Furthermore, *n* = 92 nonpediatric individuals were identified and removed from the sample, leaving *N* = 973 as the discovery sample for further algorithm development.

#### Replication Sample

The broad search criteria were deployed in the replication database (see the Study Population and Overview of Approach section) to first delineate a replication sample. The replication sample contained 13,652 pediatric records meeting the broad search criteria and was then used for validation of the performance of APT-DLD, testing whether the automated algorithm can be applied to larger databases to identify cases with the best definition of DLD. The demographics of the replication and discovery samples are presented in Table 1.

**Table 1.** Demographic characteristics of the discovery and replication samples.

Variable	Category	Discovery sample *n* (%)	Replication sample *n* (%)
Race			
	Caucasian	561 (58)	6,392 (47)
	African American	225 (23)	2,619 (19)
	Hispanic	137 (14)	1,585 (12)
	Asian	29 (3)	300 (2)
	Unknown	11 (1)	2,627 (19)
	Native American	1 (0.1)	26 (0.2)
	Other	8 (0.8)	84 (0.6)
	Multiple race	1 (0.1)	19 (0.1)
Gender			
	Male	653 (67)	9,763 (72)
	Female	320 (33)	3,884 (28)
	Unidentified	0 (0)	5 (0.04)
*N*		973	13,652

*Note.* The table shows the number of electronic health records per race/gender group in the discovery and replication samples. Percentages are denoted in parentheses.

### Manual Chart Review (Step 2)

#### Rubric Development

SLP clinicians (authors S. M. C. and K. M.) manually reviewed 100 charts randomly selected from the discovery sample. A holistic review of the charts using ICD codes, dates, and clinic notes (as in Lingren et al., 2016) was used to formulate a manual chart review rubric. The purpose of the rubric was to aid the training of research assistants (RAs) and non-SLP personnel to also manually identify DLD from EHRs. This initial qualitative review suggested that a subset of patients with a DLD-consistent phenotype had other significant medical issues unrelated to language development. For patients who had LD symptom codes but who did not have a DLD-consistent phenotype, the LD was generally due to the nature of the LD symptoms likely stemming from either comorbid developmental diagnoses inconsistent with the DLD phenotype (autism, cerebral palsy [CP]) or acquired LD (i.e., head trauma). The broad search criteria and manual review process described are summarized below and reported in more detail in Supplemental Material S1.

To untangle the complex phenotypic nature of DLD, we developed four categories to classify these EHRs, which were designed to capture separate trends in the clinical characteristics of the records with associated LD codes. EHRs in Categories 1 and 2 were classified as DLD inclusion cases because their diagnosis was not attributable to a known genetic or pervasive developmental condition (such as ASD, CP, or intellectual disability) or to TBI, stroke, or another form of acquired LD. EHRs in Category 3 (pervasive developmental condition) or Category 4 (TBI, stroke, or another acquired LD) were considered as non-DLD and excluded from the final DLD cohort because LD symptoms in these EHRs were precipitated by neurological or traumatic insults. Manual chart review criteria are described in detail in the Developmental Language Disorder Manual Chart Review Rubric in Supplemental Material S1 and also summarized below.

Category 1 (common conditions and medical encounters) describes DLD records that have no major medical conditions or trauma that may cause language impairment. Patients may have common childhood health conditions such as asthma, appendicitis, and transient otitis media (not associated with hearing loss). In addition, minor head injuries reported in the notes that did not require hospitalization or concussions that did not result in a loss of consciousness met the criteria for Category 1 placement because there was no evidence in the record that these were associated with major medical events causing permanent neural trauma.

Category 2 (significant medical conditions) describes DLD records with significant medical conditions that do not share a known genetic or neurodevelopmental basis with language impairment. Individuals in Category 2 may have codes and/or notes for mood disorders, diabetes, schizophrenia, obesity, or other major hospitalization events without head trauma or loss of consciousness such as due to kidney or heart failure, cancer, surgeries, and so forth. However, these cases were still considered to be representative of DLD, as their language impairment was not related to neurodevelopmental disorders or trauma.

Category 3 (neurodevelopmental disorders) describes cases with LD codes that also have medical, genetic, or developmental concerns known to affect neurological development. Individuals who have physiological conditions due to physical damage or delay in the physical development of their peripheral organs involved in speech and language ability also fall into this category. These biological deficits may affect and diminish perceived language ability and cause them to be erroneously classified as individuals having DLD. Epilepsy, autism, DiGeorge syndrome, Prader–Willi syndrome, cleft palate, and CP, among others, are examples of codes that fall into this category.

Category 4 (acquired LD) describes cases that have LD codes accompanied by codes for external physical and/or psychological traumas and traumatic brain injuries occurring concurrently with or prior to the first LD diagnosis in the record. Examples include stroke, child abuse, and traumatic brain injuries such as cerebral edema secondary to accident, anoxic brain damage, and concussion with loss of consciousness. Cases with trauma codes or notes occurring after the first date of DLD were classified as Category 2 as opposed to Category 4.

#### RA Training and Manual Chart Review Rubric Development

The SLP clinician coders trained authors R. N. and C. E. W. (RA coders) on the rubric, who then coded the same 100 charts previously coded by the SLP clinician coders. We analyzed concordance between our results, reconciled our differences, and refined the rubric to reflect this process. As an example, CP was consistently coded as non-DLD in all phases of development. However, there was discussion as to whether this should be coded as Category 3 or 4. On the one hand, most cases of CP arise pre- or perinatally, placing it in Category 3. On the other hand, CP could be viewed as a form of trauma on an otherwise typically developing system, placing it in Category 4. We ultimately reached consensus for coding in Category 3 because there can be instances where CP symptoms are mild and not diagnosed early enough, although the disorder is present. In such cases, a later diagnosis does not rule out the likelihood that CP impacted language acquisition early on. After iterative rounds of refining the rubric, the RA coders used the refined rubric to carry out the complete manual chart review of the 973 records. As explained above regarding chart review rubric development, this four-category system was designed to capture the main trends associated with LD symptom codes observed in this EHR database.

### Automated Algorithm Development (Step 3)

**Figure 3.**
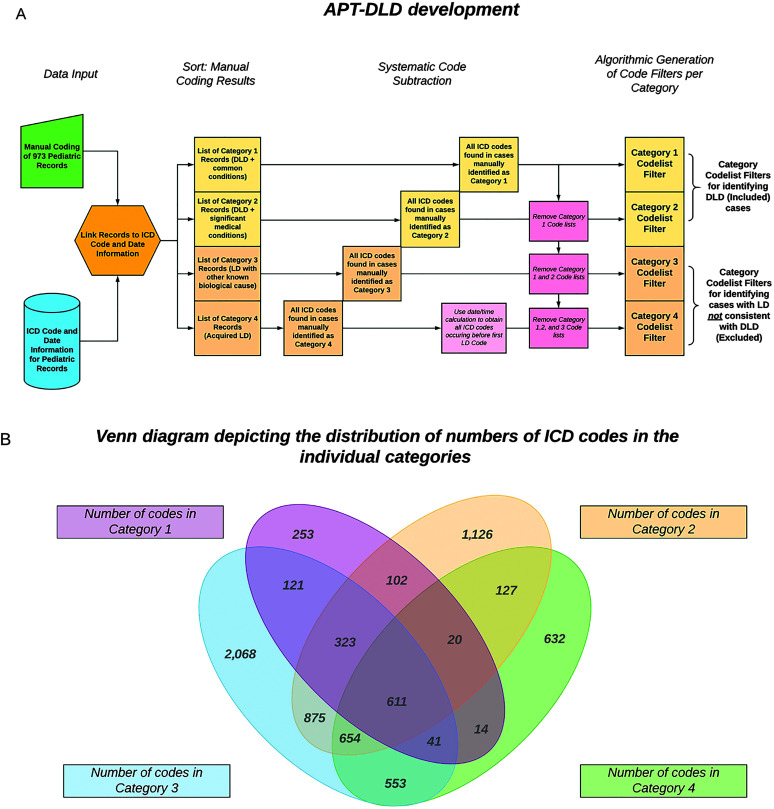
(A) An outline of the development of the category filters utilized by the Automated Phenotyping Tool for identifying DLD cases in health systems data (APT-DLD) algorithm in categorizing electronic health records is shown. The category filters were elaborated from the manually coded records of the discovery sample and formed the basis of the APT-DLD categorization system. The systematic subtraction system and the priority system for the categories that were employed by APT-DLD are also shown. (B) The systematic subtraction method used to identify category code-list filters was based on the distribution of the number of ICD (International Statistical Classification of Diseases and Related Health Problems) codes within the four categories. We found that ICD codes were not unique to each category but were shared between the categories to some extent. To identify the unique characteristic codes for each category, we assigned a priority to each category, from highest to lowest (4, 3, 2, and 1). Thus, Category 1 codes are found in all other categories, and the code list for Category 1 comprises all 1,485 ICD codes that were associated with cases manually identified from the discovery sample as Category 1. The Category 2 code list does not include any Category 1 ICD codes (Category 2 – Category 1 codes). Similarly, ICD codes that define Category 3 do not include any Category 1 or 2 codes (Category 3-2-1), and Category 4 codes comprise those left after removing codes found in all other categories. The numbers of the codes found in each of the categories and the various subsets are indicated in each cell. DLD = developmental language disorder; LD = language disorder.

**Figure 4.**
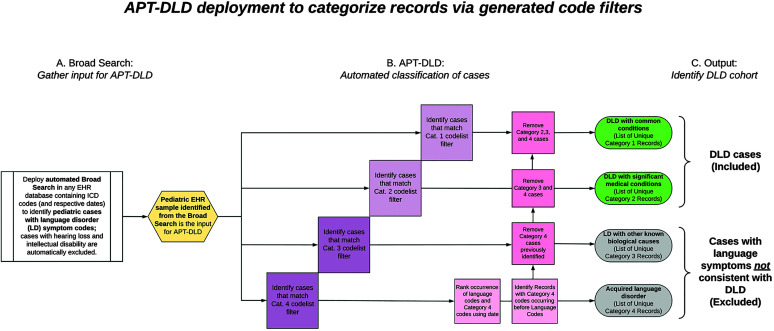
Schematic of Automated Phenotyping Tool for identifying DLD cases in health systems data (APT-DLD) deployment. APT-DLD puts electronic health record (EHR) data through the following three phases (using code freely available at https://phekb.org/phenotype/developmental-language-disorder). Phase A: A research sample is prepared using the automated broad search tools to identify pediatric records with language disorder (LD) symptom codes while excluding records with hearing loss and intellectual disability codes. Phase B: APT-DLD automatically performs categorization on EHRs using the code filters developed during the algorithm training and validation process (see the Method and Results sections). Phase C: The algorithm output consists of developmental language disorder (DLD) cases (Categories 1 and 2 are DLD with common conditions and significant medical conditions, respectively) and excluded records (Categories 3 and 4 are LD due to other biological causes or trauma). ICD = International Statistical Classification of Diseases and Related Health Problems.

We created an algorithm, APT-DLD, using open-source KNIME software (Berthold et al., 2007) to automate the manual coding procedures outlined above, which were used to identify DLD cases in the discovery sample. APT-DLD utilizes a variety of special task functions called “nodes”, which perform data access, manipulation, and analytics. APT-DLD was created using the classification results of the manually coded discovery sample to mimic the SLP clinicians' manual review process on EHRs in the discovery sample (an overview of the development of APT-DLD is shown in Figure 3A). Based on the list of ICD codes present in every record classified into each category, APT-DLD created code filters such that every ICD code listed in our discovery sample was associated with a category. For example, APT-DLD automatically determined ICD-9 Code 299.00 (i.e., autism disorder; current or active state) to be in Category 3. However, it is not reasonable to presume that any record classified into a particular category exclusively had ICD codes pertaining to that same category. APT-DLD thus used a systematic subtraction method to isolate ICD codes that truly characterized a specific category. Figure 4 illustrates the deployment of APT-DLD in a sample of EHRs identified after the broad search.

To implement this mathematical procedure, each category was first given a hierarchical level of priority: from highest to lowest priority (4, 3, 2, and 1). Thus, the presence of a Category 4 code (brain trauma) before signs of LD would supersede a Category 3 diagnosis (e.g., autism disorder diagnosis), and the patient would be binned as a Category 4 record. Similarly, if an EHR had ICD codes found in Categories 1, 2, and 3, the EHR was ultimately classified as a Category 3 case, because Category 3 takes precedence over Categories 1 and 2. This hierarchical priority system was reflected in the formation of the code filters. ICD codes in all Category 1 cases were automatically classified as Category 1 codes. The list of ICD codes found in Category 2 cases may overlap with Category 1 codes, but they also had codes *not* found in the Category 1 list. The codes unique to Category 2 were used in that category's code filter. Likewise, the list of ICD codes in Category 3 cases was refined to determine the code filter unique to Category 3; that is, codes identified in Categories 1 and 2 were removed (subtracted) to obtain the Category 3 filter. Obtaining the ICD code list for Category 4 was contingent upon the dates of the codes as well, because trauma had to precede the LD symptoms (see the Rubric Development section). Therefore, APT-DLD used a date/time calculation to identify all the codes that occurred before the first LD code in each Category 4 case. From this obtained list, previously identified Category 1, 2, and 3 codes were subtracted to obtain Category 4 codes (see Figure 3B).

APT-DLD used the code lists for each category as category filters to classify individual EHRs based on their ICD codes and dates. Identifying cases in each category also depended on the hierarchical priority level stated above. Category 4 cases were identified first, based on if EHRs had any of the ICD codes in the Category 4 filter and if these codes occurred before the first ICD language code. Then, Category 3 cases were identified by cross-referencing cases with the Category 3 filter, to obtain a list of EHRs with Category 3 codes. From these EHRs, the previously identified Category 4 cases were removed to determine EHRs classified as Category 3 cases. Similarly, Category 2 cases were identified by using the Category 2 filter and then removing the identified Category 3 and 4 cases. Finally, Category 1 cases were those cases that had Category 1 ICD codes (listed in the Category 1 filter), with the Category 2, 3, and 4 cases removed (subtracted). Note that the EHRs in both the discovery and replication samples contained an undifferentiated mix of ICD-9 and ICD-10 codes, and thus, APT-DLD utilizes code filters based only on the training sample (discovery sample); establishing the functional equivalency of additional mappings between ICD-9 and ICD-10 codes is beyond the scope of the current project. The classification system of APT-DLD was initially derived from our manual coding of the discovery sample and then automated; thus, further application of the APT-DLD algorithm in EHR systems does not require manual coding of records by the user.

### Clinician Gold-Standard Validation (Step 4)

We randomly selected 10% of the pediatric records from the *N* = 973 discovery sample to evaluate our manual chart review procedures. These 97 records were concomitantly coded by an SLP clinician coder who participated in the previous phenotyping (author S. M. C.) and an independent naïve SLP clinician coder (author C. T. B.), who applied their clinical expertise and phenotype knowledge combined with the Developmental Language Disorder Manual Chart Review Rubric created for the project (see Supplemental Material S1). We then compared the SLP clinicians' categorization results to the trained RA manual coders' results of the same 97 cases as intercoder reliability, which is reported in Supplemental Material S2 as percent agreement. The naïve SLP coder's results were also compared to APT-DLD's classification to obtain the predictive values for the algorithm for both samples.

### Validation of APT-DLD in a Replication Sample

After confirming the performance of APT-DLD in the discovery sample, we deployed it in the larger replication sample (see the Broad Search (Step 1) section for details). After APT-DLD had classified all 13,652 EHRs in the replication sample, we verified its performance in relation to the manual review of randomly selected 136 (1% of 13,652) records coded by our naïve SLP clinician coder (C. T. B.). The performance of APT-DLD is reported as the positive predictive value (PPV) of the algorithm.

## Results

### Characteristics of the Study Samples

The demographics (ethnicity, race, and gender) of the included cases (records with DLD—Categories 1 and 2) for the discovery sample (*N* = 973) and the replication sample (*N* = 13,652) as identified by APT-DLD are summarized in Table 2. Complete demographic summaries for each sample are documented in Table 1 (see the Method section).

**Table 2.** Demographics of race and gender for developmental language disorder (DLD) cases (cases in Categories 1 and 2) in discovery and replication samples as identified by the Automated Phenotyping Tool for identifying DLD cases in health systems data (APT-DLD) algorithm.

Variable	Category	Discovery sample *n* (%)	Replication sample *n* (%)
Race			
	Caucasian	248 (52)	2,320 (39)
	African American	119 (25)	1,016 (17)
	Hispanic	87 (18)	680 (11)
	Asian	13 (3)	94 (2)
	Unknown	5 (1)	1,843 (31)
	Native American	0 (0)	11 (0.2)
	Other	5 (1)	45 (0.7)
	Multiple race	0 (0)	4 (0.1)
Gender			
	Male	325 (68)	4,282 (71)
	Female	152 (32)	1,726 (29)
	Unidentified	0 (0)	5 (0.1)
*N*		477	6,013

*Note.* The numbers of electronic health records classified are shown, and percentages of the samples are indicated in parentheses.

### Manual and Automated Phenotyping of the Discovery Sample

The results of the manual and algorithmic phenotyping were as follows: According to our manual coding of the 973 records in the discovery sample, 469 EHRs met our DLD inclusion criteria (200 in Category 1 and 269 in Category 2), and 504 records met our exclusion criteria (393 in Category 3 and 111 in Category 4). APT-DLD classified 477 records into the inclusion group, of which 469 were also identified by the manual coders along with eight additional EHRs (205 in Category 1 and 272 in Category 2), and a further 496 EHRs into the exclusion group (399 in Category 3 and 97 in Category 4), all also classified in the exclusion group by the manual coders.

We then estimated the prevalence of DLD in these study samples in relation to the total numbers of pediatric EHRs found in the discovery and replication samples (using available database estimates of pediatric records from 2019). In the discovery sample, *n* = 469 DLD records (in relation to 19,176 total pediatric EHRs) account for a prevalence of 2.5%. Similarly, in the replication sample, 6,013 EHRs (in relation to 561,884 total pediatric EHRs) met the DLD criteria (1.1% occurrence).

### Intercoder Reliability

The SLP clinician coder developed manual chart review rubric is considered the gold standard, that is, our preferred method of diagnosing DLD. Our SLP clinician coders (S. M. C. and C. T. B.) coded 10% (97) of the records from our discovery sample (as stated above), the results of which were compared to the trained RA coders' review of the same charts. We achieved 93% average intercoder reliability between the SLP clinician coders' and the trained RA coders' manual chart review, indicating that our manual review was in keeping with clinical standards of DLD classification. Furthermore, the SLP clinician coders had 90% intracoder reliability between them, indicating that the manual chart review rubric was reliable for training naïve coders with and without SLP training and experience. The results of inter- and intracoder reliability for the *n* = 97 records from the discovery sample as percent agreement are summarized in Supplemental Material S2.

### Algorithm Performance Analysis

Having validated the manual review classification system of trained RA coders in relation to those of our SLP clinicians' in the previous step, we then assessed the accuracy of our algorithmic categorization of the discovery sample by comparing APT-DLD's automated classification results to the entirety of the trained RA coders' manual review results. These comparative analyses, here defined as concordance and true positive and negative rates, depend on four variables: true positives (*a*), true negatives (*b*), false positives (*c*), and false negatives (*d*) as identified by APT-DLD. We used true positive rate (TPR) and true negative rate (TNR) to determine the efficacy of APT-DLD's categorization in relation to RA coding. TPR (*a*/(*a* + *d*)) determines whether our algorithm is able to correctly classify a case as having DLD. TPR was used to compare the records that met the inclusion criteria (Categories 1 and 2) between the algorithm and trained RA coders, and it is the proportion of cases correctly classified by APT-DLD into Categories 1 and 2. TNR (*b*/(*b* + *c*)), on the other hand, indicates the probability of being classified as Category 3 or 4 when the individual has been correctly sorted to Category 3 or 4, respectively, and it determines whether our algorithm is able to accurately classify a case that matches the broad search criteria, as *not* having DLD. For the discovery sample, the TPRs for Categories 1 and 2 are 100% and 98%, respectively, whereas the TNRs for Categories 3 and 4 are 99% and 87%, respectively (see Table 3). It is important to note that, while some approaches utilize a group of control cases to compute the PPV and the negative predictive value (NPV; Nattinger et al., 2004), identification of an appropriate control typical language development cohort with no LD symptoms is outside the scope of the current project, and thus, all concordance analyses described here are situated in the context of how accurately the algorithm classifies records into DLD cases and other non-DLD LD cases (see examples in Barnado et al., 2017; Dennis et al., 2019).

**Table 3.** Results of the manual coding and Automated Phenotyping Tool for identifying Developmental Language Disorder cases in health systems data (APT-DLD) classification of electronic health records (EHRs) in the discovery sample, in terms of true positive rate (TPR), true negative rate (TNR), concordance, positive predictive value (PPV), and negative predictive value (NPV).

Group	Category	Discovery sample (*N* = 973)				
		Manual coder classification *n* (%)	APT-DLD classification *n* (%)	TPR or TNR	Concordance	Clinician gold standard
Inclusion	1	200 (21)	205 (21)	100%	98%	PPV = 95%
	2	269 (28)	272 (28)	98%		
Exclusion	3	393 (40)	399 (41)	99%	100%	NPV = 85%
	4	111 (11)	97 (10)	87%		

*Note.* Columns 3 and 4 indicate the numbers of records and percentages of the discovery sample (in parentheses) classified in each category. In Columns 5 and 7, TPR and PPV refer to the inclusion group, whereas TNR and NPV refer to the exclusion group. TPR, TNR, and concordance are calculated relative to the trained research assistant coding of the entire discovery sample. PPV and NPV are calculated with reference to the 97 EHRs (10% of the discovery sample) manually reviewed by our speech-language pathologist clinician coders, C. T. B. and S. M. C.

We also analyzed concordance for our overall inclusion and exclusion groups. For inclusion, concordance (defined as: *a*/(*a* + *c*)) is used to compute the percentage of cases that were included (Categories 1 and 2) in both the algorithm results and the manually coded results. We similarly calculated the concordance for our exclusions (defined as: *b*/(*b* + *d*)), which computed the percentage of records that were excluded (Categories 3 and 4) in both the algorithm results and the trained RA manually coded results. A high concordance for either inclusion or exclusion criteria indicates that the performance of the automated implementation of APT-DLD is accurately mimicking the manual chart review, which is what we set out to achieve. The concordance for the inclusions is 98%, and that for the exclusions is 100%. The algorithm also achieved 98% overall correctness; that is, it correctly identified and categorized 949 records out of 973. We further compared the results of the 97 EHRs from the discovery sample that were independently coded by our naïve SLP clinician coder (author C. T. B.) to APT-DLD's classification in order to ascertain the PPV and the NPV. Table 3 documents the results of the analysis between APT-DLD and the trained RA and SLP clinician coders for the discovery sample.

In the replication sample, the SLP clinician coder (C. T. B.) also manually coded 136 EHRs (see the Method section). The PPV for the replication sample in relation to the SLP clinician coder is 90%. The results of the deployment of APT-DLD in the replication sample are reported in Table 4. The NPV for the replication sample was 64%, which indicates that APT-DLD identifies few false positives but comparatively more false negatives.

**Table 4.** Results of Automated Phenotyping Tool for identifying Developmental Language Disorder cases in health systems data (APT-DLD) classification when deployed in the replication sample as well as subsequent positive predictive value (PPV) and negative predictive value (NPV) in relation to inclusion and exclusion decisions from manually reviewed charts by a speech-language pathologist clinician coder (*n* = 136; 1% of the total replication sample *N* = 13,652).

Group	Category	Replication sample	
		APT-DLD algorithm classification *n* (%)	Clinician gold standard
Inclusion	1	3,030 (22)	PPV = 90%
	2	2,983 (22)	
Exclusion	3	6,729 (49)	NPV = 64%
	4	910 (7)	

*Note.* PPV refers to the inclusion group, whereas NPV refers to the exclusion group.

Our review of post-algorithm data, from the replication sample, showed that the few false-positive EHRs that were detected by APT-DLD had SLP notes that mentioned either premature birth or developmental issues not notated using ICD codes. The larger number of false negatives (26) was a result of codes that APT-DLD recognized as Category 3 or 4, due to the automation employed during the development of the algorithm in the training set. In particular, 10 EHRs had codes for ocular disorders, unspecified learning disorder codes, and developmental codes that are sometimes associated with neurodevelopmental disorders such as CP and autism. Similarly, three EHRs had fracture codes in the absence of neural trauma; since traumatic events are a feature of Category 4, it appears that some other codes associated with trauma were learned by the algorithm. Other misclassifications included EHRs that had codes associated with prematurity or codes correlated with other developmental disorders that affect language. Since the category code lists are derived from the distribution of ICD codes for all EHRs, there is a chance that certain nonessential codes would be included in categories by virtue of simple association with category-defining codes such as those for neurodevelopmental issues, trauma, and so forth. In line with this, APT-DLD also excluded EHRs based on miscellaneous minor symptoms that may be associated with more serious conditions in the training set but are not considered exclusionary by themselves. By demonstrating high overall correctness for APT-DLD classification, we show that diagnostic codes do, to a large extent, capture relevant clinical information and can be used to build data mining algorithms. Future development and validation of the APT-DLD algorithm could seek to decrease false-negative rates by applying criteria iteratively. Taken together, these results indicate that the algorithm identifies instances of DLD from EHRs successfully, although at the cost of a miss rate that causes some cases to be incorrectly rejected and, thus, renders the algorithm as conservative.

To summarize, we developed an open-source automated algorithm that successfully mimics manual chart review for classifying patients' EHRs as DLD cases (vs. non-DLD), in an initial sample of nearly 1,000 EHRs. We validated APT-DLD's automated classification in relation to independently conducted SLP clinician chart reviews with a PPV of 95%. We then applied APT-DLD to a much larger (separate) sample of over 13,000 pediatric records, and the algorithm achieved a 90% PPV for the classification of DLD cases, indicating validity and scalability for DLD cohort identification in large-scale health systems data.

## Discussion

Epidemiological approaches to speech and language disorders have the potential to provide new insights into the causes, risks, treatments, and outcomes at the population level (Raghavan et al., 2018; The National Academies of Sciences, Engineering, and Medicine, 2016). Identification and cohort selection for developmental speech and language disorders within large EHR databases represent an area for potential growth that has been little explored. Notably, for multi-etiological syndromes such as DLD, it has in the past been time consuming and complicated to conduct a systematic chart review to achieve a well-defined phenotype for cohort identification. However, recent works on autism (Bush et al., 2017; Leroy et al., 2018; Lingren et al., 2016), mental illness (Lyalina et al., 2013; McCoy et al., 2018), and even infectious diseases (Chiu & Hripcsak, 2017) demonstrate the feasibility of applying automated algorithmic procedures to EHRs for phenotyping of complex diseases and disorders. Here, we developed, evaluated, and validated a rule-based algorithm, APT-DLD, that automatically classifies cases of DLD from EHRs, to address the challenge of identifying large cohorts of DLD within health systems. This is, to our knowledge, the first study to apply a data mining algorithm to EHR databases for identifying DLD and represents an overture to big data EHR research in the field of communication disorders.

A major component of the algorithm development process was to first create and validate a DLD manual chart review rubric, under the guidance of SLPs, that allowed trained coders to determine the DLD phenotype among EHRs retrieved from a broad search for LD symptoms (see Supplemental Material S3 and the Method section). A significant aspect of manual coding was ascertaining the hallmarks of a DLD phenotype within the EHRs using a holistic review process that incorporated information from ICD codes and clinical notes. The coding system distinguished these cases of DLD from other cases of LD symptoms caused by other conditions (i.e., other neurodevelopmental disorders or acquired LDs; see Supplemental Material S1). The chart review rubric for DLD served as a guide to assist novice coders in differentiating these phenotypes. Encouragingly, high coder reliability between our RA manually coded set and the clinicians' gold standard of coding was obtained (our SLP clinical experts coded 10% of our discovery sample, and intercoder reliability between the trained RA coders and the SLPs was 89% for DLD inclusion decisions and 97% for exclusion decisions), adding further confidence to the design of EHR-based phenotyping principles for DLD.

We then automated this classification procedure and created a new tool called “APT-DLD” to perform classification on EHR data. The classification system and the results of the chart review of nearly 1,000 EHRs in our discovery sample acted as the basis for automated algorithm development. Specifically, the classifications of the discovery sample were employed as a benchmark for phenotype identification and automation, such that the categorization of each record from manual chart review was used to delineate relatively stable criteria for the inclusions and exclusions. The inclusions (Categories 1 and 2) can be reasonably expected to define a DLD phenotype, that is, records of patients who have a higher chance of being diagnosed with bona fide DLD, whereas the exclusions (Categories 3 and 4) are cases that have an interfering biological or external factor that may be directly responsible for their LD diagnosis exclusive of DLD. By differentiating the cohort into these two broad groups, we identified a large set of universal ICD codes that provide a phenotypic definition for DLD and a set of factors that may be associated with a DLD diagnosis (Anderson et al., 2016).

One caveat to our process may be that EHRs often include limited qualitative data, which makes modeling and testing of the accuracy of phenotyping crucial (Hripcsak & Albers, 2013). Thus, in the initial applications of our EHR phenotyping algorithm, output must be compared to the SLP clinician phenotype characterization of these records. Modeling the function of APT-DLD on the manually reviewed discovery sample thus provided a strong basis for validating the results of the algorithm. As our phenotype for DLD is defined by ICD codes and dates that are universal features of EHRs, APT-DLD can be adapted for use across any other EHR database that utilizes ICD codes and dates of code entry as part of patient records. We applied APT-DLD to an additional set of records in a nonoverlapping replication sample of *N* = 13,652 pediatric records containing LD symptom codes (drawn from VUMC's Synthetic Derivative database) and, from that sample, successfully classified 6,013 DLD cases. This application of the algorithm achieved a 90% PPV for the identification of DLD cases (in relation to gold-standard SLP clinician coding), thus demonstrating APT-DLD's versatility. This PPV is in line with the reliability reported in other automated phenotyping algorithm studies (Lingren et al., 2016; Paul et al., 2018).

Furthermore, in both our discovery and replication samples, the prevalence of DLD was estimated to be approximately 1.8%. Accounting for the fact that population-wide prevalence is 7% (Tomblin et al., 1997) and that nearly three fourths of DLD cases are not identified, this 1.8% occurrence level in EHRs appears to coincide with the expected identification rate of actual DLD cases in the population. Given that the clinical definition of DLD we used here is less restrictive than, but still inclusive of, the classical definition of SLI, we may assume, based on statistical likelihood, that the DLD cohort identified here in fact includes many SLI cases, responding to recent calls to augment the clinical relevance of DLD/SLI research based on the more expansive DLD definition (McGregor et al., 2020).

Still, one important limitation of our study is the inability to access individual IQ scores, language scores, grammar tests, and educational outcome measures from the EHR database. This is a common limitation in many public health records (Raghavan et al., 2018). Of course, when available, these details provide a further refined behavioral phenotype (Lancaster & Camarata, 2019), which would bring greater specificities to the inferences one can draw regarding the impact of DLD in a cohort (Evans et al., 2015; Laasonen et al., 2018; Newbury et al., 2011; SLI Consortium, 2002). However, most EHRs do not carry the more focused phenotypic descriptors such as diagnostic test information. Thus, our endeavor uses common and widely available ICD denominators in EHRs to scale up our identification of DLD in the population. As Raghavan et al. (2018) report, even public health databases limited exclusively to diagnostic classification can be highly useful for phenotyping when validated, as was done herein. It is also important to note that both our discovery and replication samples were mined from VUMC's database, thus restricting our population geographically. Deploying this algorithm across various sites will also broaden the demographic base of the identified cohorts. It is important to note that, because our phenotype was developed from definitions of DLD used primarily in English-speaking countries (see Bishop et al., 2016), this procedure may need additional validation when applied to EHRs in other international health systems. Finally, individuals who did not meet the broad search criteria may not represent true controls because they may have language impairments (DLD or otherwise) not recorded in their EHR. However, developing a similar algorithm to identify these control groups (e.g., for additional phenotypic or genetic investigations) will be a separate endeavor and will depend on the type and quality of information available in EHRs.

Our base phenotype (in the manually reviewed discovery sample) was derived from a holistic chart review of all the information present in each patient's EHR (including clinical notes and ICD codes), which would not have been possible if these clinical notes were not available in our EHR database. The variability in the presence of SLP referrals and definitive diagnoses describes our diverse set of cases that met our definition for inclusion (DLD classification) and highlights the need for efficient identification of LDs (see Supplemental Material S3). Other recently developed EHR data mining algorithms employ natural language processing to automatically classify phenotypes using text recognition from medical notes and other clinical information (Albers et al., 2018; Deferio et al., 2018; Wei et al., 2016). APT-DLD, however, is automated, is rule based, and does not depend on natural language processing or access to medical notes. Thus, although the information from clinical notes initially helped us develop our coding rubric and was used in manual chart review, such information is not applied or required in the automated algorithm. Latent information from the clinician notes is correlated with ICD codes; helped us define the DLD phenotype; and, importantly, was captured by the algorithm, even though the notes are not a component of the algorithm. This underlying relationship was reflected in the very high correspondence between the manual and automated classifications of records in both our discovery and replication samples. It is important to recognize the versatility of applying a rule-based algorithm such as APT-DLD, which is not restricted by language (because clinical notes are not utilized), to other databases and biorepositories.

It is important to note that the automated functioning of APT-DLD and simple computational implementation with open-source software make it possible to rapidly apply this algorithm to thousands of EHRs at once, which would not be possible with a method that depends entirely on manual chart review. The scale of this new tool thus can enable a new wave of epidemiological investigation, identifying much larger cohorts of DLD and advancing our understanding of DLD through better powered comorbidity analyses (i.e., phenome-wide association studies or clustering analyses; see Lingren et al., 2016; Prieto et al., 2016; Prinzbach et al., 2018). We believe that APT-DLD will be able to provide knowledge on health/biomedical factors linked to DLD that is complementary to other valuable demographic, language, and educational risk factors associated with LDs gained from modeling approaches carried out in an educational context (Justice et al., 2019). Importantly, our EHR-based algorithm approach also opens future opportunities for genome-wide association studies in EHRs linked to genetic data and may complement prior genome-wide association studies with smaller sample sizes (usually within communities and families) of the DLD phenotype (Deriziotis & Fisher, 2017; Evans et al., 2015; Kornilov et al., 2016).

APT-DLD serves as a potentially valuable tool in harnessing the ICD code information from EHRs for DLD, a highly prevalent but understudied disorder (The National Academies of Sciences, Engineering, and Medicine, 2016; Tomblin et al., 1997), and may have broader applicability to international databases and toward building large samples for genomic discovery. Future studies should focus on applying APT-DLD to other EHR data sets in order to further refine the phenotype as captured in EHRs, widen the demographic base, improve our understanding of the risk factors and etiology of DLD, and generate large samples for phenome–genome investigations (Anderson et al., 2016; Lingren et al., 2016).

## Supplementary Material

10.1044/2020_JSLHR-19-00397SMS1 Supplemental Material S1 Developmental language disorder (DLD) manual chart review rubric.

10.1044/2020_JSLHR-19-00397SMS2 Supplemental Material S2 Intercoder reliability for research assistant coders and SLP coders for 10% of the discovery sample.

10.1044/2020_JSLHR-19-00397SMS3 Supplemental Material S3 Determining the DLD phenotype among EHRs retrieved from a broad search for LD symptoms.
