# Supplementary material for: Automated Phenotyping Tool for Identifying Developmental Language Disorder Cases in Health Systems Data (APT-DLD): A New Research Algorithm for Deployment in Large-Scale Electronic Health Record Systems
Source: J Speech Lang Hear Res. 2020 Aug 11;63(9):3019–35. doi: 10.1044/2020_JSLHR-19-00397 (PMC7890229; doi:10.1044/2020_JSLHR-19-00397)
Supplement: Supplemental Material S1 [file JSLHR-63-3019-s001.pdf]

Supplemental Material S1. Developmental language disorder (DLD) manual chart review rubric.

This rubric describes the design of the DLD phenotyping process for EHRs, including the broad search criteria and how to categorize them manually during chart review.

Broad Search Criteria:

These are the basic inclusion and exclusion codes that are used to generate the primary dataset for the study.

Inclusion codes: Presence of a single occurrence of any one of the following ICD codes are indicative of language disorder (LD) symptoms and define the broad search criteria:

ICD-9 codes	ICD-10 codes
315.39 (Other developmental speech or language disorder)<br>315.32 (Mixed receptive-expressive language disorder)<br>315.31 (Expressive language disorder)	F80.89 (Other developmental disorders of speech and language)<br>F80.2 (Mixed receptive-expressive language disorder)<br>F80.1 (Expressive language disorder)

These 6 codes are referred to as "LD codes" throughout the rubric.

Note: Code F80.9 is not used as an LD code, given that SLPs clinicians do not regularly use this code to identify language disorders or DLD. Rather, this is an "unspecified" code used primarily in cases where the clinician or SLP does not have enough clinical information for make a formal diagnosis, the etiology of the language impairment is unclear, or the issue is transient and has an external trigger. Since our focus lies on classifying already-diagnosed LD or DLD cases, we have not included this code in our LD definition (see American Speech-Language-Hearing Association [ASHA], 2019).

Records containing a single occurrence of any of the following codes cause records to be excluded from the broad search:

ICD9 code group 389 – Hearing loss
ICD9 code 317 – Mild intellectual disabilities
ICD9 code group 318 – Other specified intellectual disabilities
ICD9 code 319 – Unspecified intellectual disabilities
ICD9 code group 758 – Chromosomal anomalies
ICD10 code group H90 – Conductive and sensorineural hearing loss
ICD10 code group H91 – Other and unspecified hearing loss
ICD10 code F70 – Mild intellectual disabilities
ICD10 code F71 – Moderate intellectual disabilities
ICD10 code F72 – Severe intellectual disabilities
ICD10 code F73 – Profound intellectual disabilities
ICD10 code F78 – Other intellectual disabilities
ICD10 code F79 – Unspecified intellectual disabilities
ICD10 code groups Q90-Q99 – Chromosomal abnormalities, not elsewhere classified (Down syndrome; Trisomy 18 and 13; Other trisomies and partial trisomies of the autosomes, not elsewhere classified; Monosomies and deletions from the autosomes, not elsewhere classified; Balanced rearrangements and structural markers, not elsewhere classified; Turner's syndrome; Other sex chromosome abnormalities, female phenotype, not elsewhere classified; Other sex

chromosome abnormalities, male phenotype, not elsewhere classified; Other chromosome abnormalities not elsewhere classified)

Non-pediatric cases:
An age filter is used to remove records where the first LD code(s) is non-pediatric (occurs when the patient is 18 years of age and above).

Category Classification:
This is the manual coding system that is used to guide classification of records as one of four categories to identify gold standard DLD. This system allows for distinction of DLD co-occurring without or with co-morbid medical conditions, Categories 1 and 2 respectively, both of which will be *included* in the study. It also identifies patients that show significant alternative (non-DLD) medical explanations for their language disorder that fall outside the DLD phenotype (Categories 3 and 4), which are then marked for exclusion.

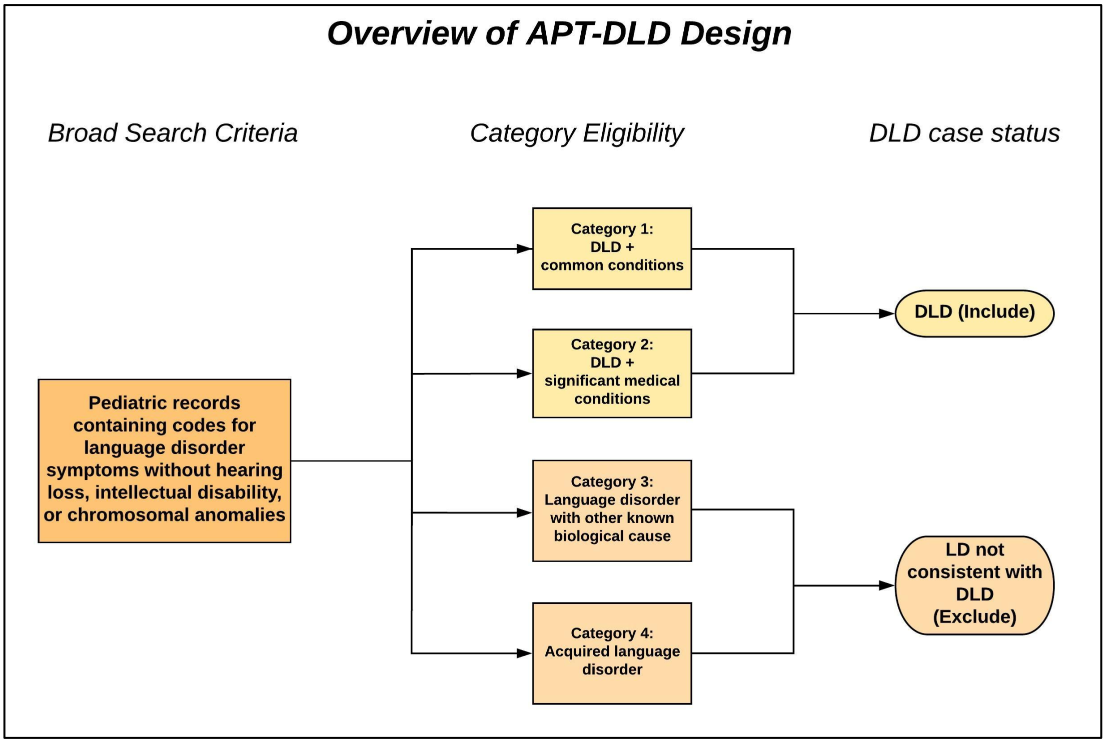

EHR phenotyping approach and design of APT-DLD, including the broad search criteria that was used to identify a sample from the EHR database and subsequent characteristics of the records which informed their classification into different typologies depending on the observed cause of the language disorder symptoms.

The manual coder examines the EHR and specifically accounts for ICD diagnostic codes, speech language pathologist (SLP) notes, and pediatrician notes in order to classify the patient as belonging to one of the following four categories:

- **Category 1— DLD not accompanied by other major medical/traumatic concern:**
  - The patient has DLD with no other major medical condition that could be causing DLD. Normal childhood conditions are acceptable.
    - Asthma, appendicitis, ADHD, ADD, oppositional defiant disorder and dyslexia are not exclusionary (they can occur in Category 1).
    - Minor head injuries that do not require hospitalization, concussions that do not include loss of consciousness, and contusions of the head are acceptable.
    - Individuals with phonological disorders can fall into this category.
      - It is important to note that although ICD10 has a separate code for phonological disorder (F80.0), in ICD9 it receives the 315.39 code which is part of our primary inclusion criteria.
    - ICD codes for eustachian tube disorders not accompanied by other anomalies of speech producing organs can fall under Category 1.
    - Tongue-tie and Dysphagia are not thought to affect language acquisition and can fall under this category.
    - Occurrences of plagiocephaly and congenital anomalies of skull and face bones are also not considered to cause language disorders or are the result of broader conditions that do not impact language.

- **Category 2—DLD co-occurring with other major medical conditions:**
  - The patient has an ongoing medical condition/health concern, or a disorder not typical of childhood, that is not thought to share a genetic basis with DLD or be related to DLD (i.e. not neurodevelopmental).
    - Acceptable co-morbid conditions under this category include:
      - Conditions that may require hospitalization such as kidney failure, congenital pulmonary and circulatory disorders, and cancer.
      - Cleft lip (without palatal clefting), minor anomalies of tongue, larynx and trachea, resonance or voice disorders.
      - Medical obesity
      - “Other conditions of brain” as an ambiguous code not related to language acquisition.
      - Mood disorders such as bipolar disorder, anxiety disorder, major depressive disorder, depression, depressive episodes, other mood disorders, suicidal and homicidal ideations, and eating disorders.
      - Schizophrenia (SCZ) and schizoid disorder.
        - Although SCZ is considered a neurodevelopmental disorder, its onset is usually not until ages 18 – 25. Thus, the manifestation of clinical symptoms would not be causal for DLD. Note though that there could be pleiotropic genetic effects that predispose people to both.
    - Genetic disorders that are not neurodevelopmentally linked to DLD, and may or may not constitute a major medical condition (i.e. polydactyly, PKU, genetic anemias) can fall under this category.

- **Category 3—Language Disorder with a known biological cause:**
    - The patient has a medical/genetic condition or developmental disorder that may affect neurological development, such as autism, and is likely causing their LD.
    - Physical damage or simple delay in physical development to peripheral organs involved in speech and language would also place a participant in this category.
        - Comorbid conditions in this category include:
            - Artificial pharynx, cleft palate, craniosynostosis.
            - Epilepsy, infantile cerebral palsy, autism (pervasive developmental disorder specified or not otherwise specified), encephalopathy, cerebral cysts, meningitis, and premature infants.
            - Di George Syndrome and Prader Willi Syndrome, and any other chromosomal anomaly not excluded in the broad search criteria.
            - Fetal alcohol syndrome and infants born with drug dependency are classified in this category as well.

- **Category 4—Acquired language disorder:**
    - Patients in this category have a trauma that affects the brain or peripheral organs involved in language, occurring before the first LD code. This could have caused the impairment or resulted in developmental delay. Instances of such trauma include:
        - Child abuse, PTSD, head injury resulting in hospitalization and psychological trauma such as diagnosis of mental illness before the onset of DLD.
        - Concussions resulting in loss of consciousness and intracerebral hemorrhaging.
        - Traumatic brain injury such as cerebral edema, intracerebral hemorrhage, anoxic brain damage, compression of brain, cerebral infarction, hypoxic ischemic encephalopathy, and obstructive hydrocephalus.
        - Any instance of poisoning, toxic effects of substances, or adverse effects of drugs prior to language codes also falls under Category 4.

Categorization depends on the relative interference of existing medical conditions that might impact the etiology of DLD. Classification preference for each record is in the order: 4, 3, 2, 1. Thus, presence of a Category 4 code (brain trauma) before signs of LD would supersede a Category 3 diagnosis (e.g. autistic disorder diagnosis), and the patient would be binned as a Category 4 record.
