# Supplementary material for: Automated Phenotyping Tool for Identifying Developmental Language Disorder Cases in Health Systems Data (APT-DLD): A New Research Algorithm for Deployment in Large-Scale Electronic Health Record Systems
Source: J Speech Lang Hear Res. 2020 Aug 11;63(9):3019–35. doi: 10.1044/2020_JSLHR-19-00397 (PMC7890229; doi:10.1044/2020_JSLHR-19-00397)
Supplement: Supplemental Material S2 [file JSLHR-63-3019-s002.pdf]

https://doi.org/10.1044/2020_JSLHR-19-00397

Supplemental Material S2. Intercoder reliability for research assistant coders and slp coders for 10% of the discovery sample.

Variable	RA coder	SLP coder 1 (SC)	SLP coder 2 (CB)
Included EHRs (Category 1&2)	48	52	52
Excluded EHRs (Category 3&4)	49	45	45
Intercoder Reliability			
% Agreement (included EHRs)	-	90%	88%
% Agreement (excluded EHRs)	-	98%	96%
% Agreement (total)	-	94%	92%
Intra-coder Reliability<br>% Agreement (included EHRs)	-	-	90%
% Agreement (excluded EHRs)	-	-	89%

Note. Values indicate the number of records classified as Category 1 or 2 (Included) or Category 3 or 4 (Excluded) out of the 97 EHRs manually reviewed (N=97). Speech Language Pathologist (SLP) Coder 1 is author SC, and SLP Coder 2 is author CB. Research Assistant (RA) coder is the final agreement of coding between authors CEW and RN. EHR = Electronic Health Record. Intra-coder reliability between the two SLP Clinician Coder uses SLP Coder 1 (author SC) as the gold standard to which author CB's coding is compared.
