# Supplementary material for: Automated Phenotyping Tool for Identifying Developmental Language Disorder Cases in Health Systems Data (APT-DLD): A New Research Algorithm for Deployment in Large-Scale Electronic Health Record Systems
Source: J Speech Lang Hear Res. 2020 Aug 11;63(9):3019–35. doi: 10.1044/2020_JSLHR-19-00397 (PMC7890229; doi:10.1044/2020_JSLHR-19-00397)
Supplement: Supplemental Material S3 [file JSLHR-63-3019-s003.pdf]

https://doi.org/10.1044/2020_JSLHR-19-00397

Supplemental Material S3. Determining the DLD phenotype among EHRs retrieved from a broad search for LD symptoms.

In the Discovery cohort, 47% of patients (220 records) in the inclusion group had documented SLP notes in their charts, such that they were either seen or referred to an SLP as recorded in the notes. Further, 21% of records in the inclusion group had notes from an SLP for a specific diagnosis of language disorder that mentioned one or more of the following ICD codes: F80.1, F80.2, 315.31, and 315.32. Another 32% of the inclusion records had multiple language disorder codes present at various dates in their patient record or a single ICD code for 315.31/F80.1 or 315.32/F80.2, but were not referred to an SLP. Lastly, 47% of the records had multiple language disorder codes for non-specific language and speech disorders (F80.89 and/or 315.39), either without notes from an SLP or had a formal SLP diagnosis for “articulation disorder,” “phonological disorder,” and/or other non-language disorders. The variability in the presence of definitive diagnosis found in the EHRs, highlight the need for better characterization and identification of DLD in medical records.
